# A large mural nodule in branch duct intraductal papillary mucinous adenoma of the pancreas: a case report

**DOI:** 10.1186/s40792-014-0009-x

**Published:** 2015-02-24

**Authors:** Koichiro Haruki, Shigeki Wakiyama, Yasuro Futagawa, Hiroaki Shiba, Takeyuki Misawa, Katsuhiko Yanaga

**Affiliations:** Department of Surgery, The Jikei University School of Medicine, 3-25-8, Nishi-Shinbashi, Minato-ku, Tokyo 105-8461 Japan

**Keywords:** Intraductal papillary mucinous neoplasm, Pancreas, Branch duct, Mural nodule

## Abstract

Indications for resection of branch duct intraductal papillary mucinous neoplasms (IPMNs) remain controversial because of their low tendency to be malignant. Surgical resection should be recommended if any factors indicating malignancy are present. However, preoperative differentiation between benign and malignant tumors is very difficult, especially in cases of branch duct IPMNs. We herein report a case of branch duct intraductal papillary mucinous adenoma (IPMA) of the pancreas with a large mural nodule of 25 mm. A 74-year-old woman was admitted for examination and treatment for a cystic tumor in the head of the pancreas. Magnetic resonance cholangiopancreatography and computed tomography showed a cystic lesion, 50 mm in diameter, with an irregular mural nodule in the pancreatic head. Endoscopic ultrasonography demonstrated a multicystic tumor connected with the main pancreatic duct (MPD). The mural nodule had a diameter of 18 mm, and the MPD had a slight dilation of 6 mm. These findings suggested a high potential for malignancy. The patient underwent pancreaticoduodenectomy with lymph node dissection. The excised pancreas showed multiple cysts located in the branch pancreatic duct with a maximum diameter of 75 mm. The mural nodule had a maximum diameter of 25 mm. The tumor was diagnosed as an IPMA by pathological examination. After operation, the patient was discharged without any complications. Two years after resection, the patient remains in remission with no evidence of tumor recurrence.

## Background

Detection of intraductal papillary mucinous neoplasms (IPMNs) of the pancreas has been increasing due to recent advances in imaging. IPMNs can be malignant and undergo transformation from an adenoma to invasive carcinoma. International consensus guidelines from 2006 [1] and recently updated in 2012 [2] recommend surgical resection of main duct IPMNs due to a high risk of malignancy, ranging from 60% to 100%. On the other hand, branch duct IPMNs have lower rate of malignancy (6% to 51%) [3-5], although surgical resection should be recommended if any factors indicating malignancy are present. Large mural nodules are associated with a higher risk of malignancy. However, differentiating between intraductal papillary mucinous adenoma (IPMA) and intraductal papillary mucinous carcinoma (IPMC) is often difficult, especially in branch duct IPMNs. We herein report a case of branch duct IPMA of the pancreas with a large mural nodule. To the best of our knowledge, this case involves the largest mural nodule diagnosed as branch duct IPMA reported so far in the English literature.

## Case presentation

**Figure 1:**
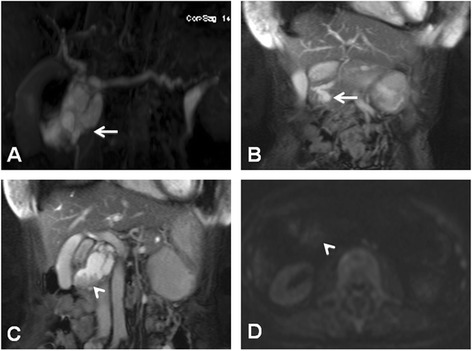
**Magnetic resonance cholangiopancreatography.** Magnetic resonance cholangiopancreatography revealed a cystic lesion located in the pancreatic head **(A, B)** (arrow) with a mural nodule **(C)** (arrow head), seen as a slight increase in intensity on diffusion-weighted images **(D)** (arrow head).

**Figure 2:**
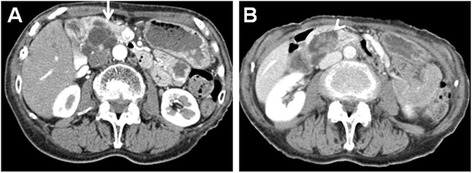
**Enhanced computed tomography.** Computed tomography showed a cystic lesion with a diameter of 50 mm **(A)** (arrow) and an irregular mural nodule, which showed gradual enhancement on enhanced CT **(B)** (arrow head).

**Figure 3:**
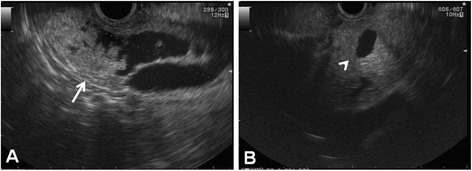
**Endoscopic ultrasonography.** Endoscopic ultrasonography demonstrated a multicystic tumor connected with the main pancreatic duct (MPD). The mural nodule had papillary growth with a diameter of 18 mm **(A)** (arrow), and the MPD had a slight dilation of 6 mm **(B)** (arrow head).

**Figure 4:**
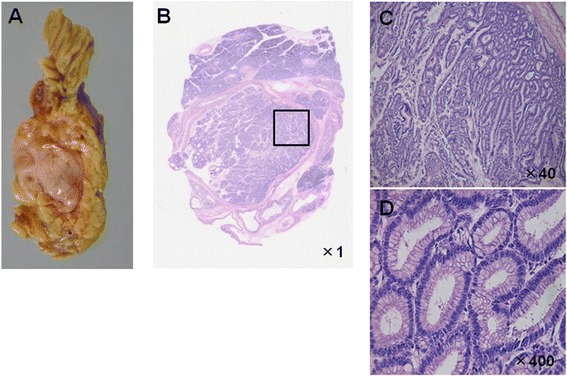
**Macroscopic and pathological findings.** The excised pancreas showed multiple cysts located in the branch pancreatic duct with total dimensions of 75 × 45 × 33 mm in the pancreas head. The mural nodule was 25 × 20 × 18 mm in size **(A)**. Pathological examination revealed that it was composed of papillary structures consisting of mucin-containing columnar epithelial cells with low-grade atypia **(B-D)**.

A 74-year-old woman presenting with epigastralgia was admitted to our hospital for examination and treatment for a cystic tumor in the pancreatic head. Laboratory data showed slightly increased levels of P-type serum amylase (57 U/l) and elastase-I (680 ng/dl). We also measured the tumor markers carcinoembryonic antigen (CEA, 3.7 ng/ml), carbohydrate antigen 19–9 (14 U/ml), and DUPAN-2 (25 U/ml). Magnetic resonance cholangiopancreatography (MRCP) revealed a cystic lesion located in the pancreatic head (Figure 1A,B) with a mural nodule (Figure 1C), seen as a slight increase in intensity on diffusion-weighted images (Figure 1D). Computed tomography (CT) showed a cystic lesion, 50 mm in diameter (Figure 2A), with an irregular mural nodule, which showed gradual enhancement on enhanced CT (Figure 2B). Endoscopic ultrasonography (EUS) demonstrated a multicystic tumor connected with the main pancreatic duct (MPD). The mural nodule had papillary growth with a diameter of 18 mm (Figure 3A), and the MPD was slightly dilated to 6 mm (Figure 3B). These findings suggested malignant potential. The patient was diagnosed with branch duct IPMC of the pancreas and underwent pancreaticoduodenectomy with lymph node dissection. The excised pancreas showed multiple cysts located in the branch pancreatic duct with total dimensions of 75 × 45 × 33 mm in the head of the pancreas. The mural nodule was 25 × 20 × 18 mm in size (Figure 4A). Pathological examination revealed a composition of papillary structures consisting of pancreatobiliary-type mucin-containing columnar epithelial cells with low-grade atypia (Figure 4B,C,D). These tumor cells were negative for p53 on immunohistochemistry. The tumor was diagnosed as an IPMA. The patient was discharged 14 days post-operation without any complications. Two years after resection, the patient remains in remission with no evidence of tumor recurrence.

## Discussion

IPMNs are an increasingly recognized entity representing a spectrum of benign and malignant neoplasms of the pancreas. While there is a general consensus that all main duct IPMNs should be resected, the indications for resection of branch duct IPMNs remain controversial because of their lower tendency for malignancy. The guidelines include a flowchart covering the suggested surgical resection and follow-up procedures for branch duct IPMNs. Recent guidelines [2] recommended that surgical resection should be considered without further testing if a patient with a cystic lesion of the pancreas has obstructive jaundice, an enhancing solid component within the cyst, or dilation of the MPD to ≥10 mm. According to the most recent guidelines, surgical resection was therefore required in this case due to the presence of an enhanced mural nodule. Moreover, older guidelines [1] recommended that resection should be performed if any of the following five factors are present: a cyst >3 cm in diameter, mural nodules, MPD dilation to >6 mm, positive cytology, or symptoms attributable to the tumor. Nagai et al. [6] evaluated the usefulness of these guidelines in branch duct IPMN and reported a high sensitivity (97.3%) but low specificity (29.8%) for predicting malignancy in branch duct IPMN according to these guidelines. Furthermore, as patients presented with more factors, there was an increase in the specificity and positive predictive value but a decrease in sensitivity and negative value of the guidelines for making a preoperative diagnosis of malignancy. Although preoperative imaging, such as EUS and MRCP, showed that the case presented here had four of the factors described in the previous guidelines, histology indicated that the tumor was an IPMA.

Some meta-analyses have evaluated the risk of malignancy. For both main duct and branch duct IPMNs, a cyst size >3 cm, presence of a mural nodule, MPD dilation, and main duct IPMNs were associated with an increased risk of malignancy [7]. For branch duct IPMNs, presence of a mural nodule, MPD dilation, thick septum/wall, and a cyst size >3 cm were indicators of malignancy [8].

Several recent studies reported that the size of mural nodules was a more significant malignant factor than tumor size for predicting the malignancy of branch duct IPMNs. For the prediction, the cutoff values for mural nodule size were 5 to 10 mm [9-12]. In addition, the MPD size, high serum CEA and carbohydrate antigen 19–9 levels, and high CEA levels in the pancreatic juice have been reported to be predictive factors for malignancy of branch duct IPMNs [11,13,14]. However, it is still impossible to detect all malignant cases, even after examination for these factors and the use of recent imaging modalities. EUS is very useful for identifying small lesions in the pancreas and a helpful modality for the diagnostic evaluation of branch duct IPMNs [12,15]. Furthermore, the mural nodule size of branch duct IPMNs detected using EUS was a reliable predictive factor for malignancy [12]. Magnetic resonance imaging is also well suited for the detection of pancreatic lesions, including IPMNs [16]. There have also been meta-analyses on these tools for differentiating malignant and benign IPMNs. Cytology based on endoscopic retrograde cholangiopancreatography was reported to have a high specificity (97.2%) but a poor sensitivity (35.1%) for distinguishing benign IPMNs from malignant IPMNs [17]. The level of cyst fluid CEA was mostly ineffective in differentiating malignancy; sensitivity was 65%, while specificity was 66% [18]. In histology, expression of human telomerase reverse transcriptase was strongly associated with malignant transformation in IPMNs [19].

In the present case, we diagnosed branch duct IPMN as a malignant tumor using several findings suggesting a high possibility of malignancy. We reviewed recent studies and summarized the mural nodule size of benign IPMNs in Table 1. To the best of our knowledge, our case is the largest reported mural nodule in branch duct IPMA. Here, the operation was justified due to a high risk of malignancy.

**Table 1:** **Reported size of mural nodules in branch duct IPMC and IPMA**

**Author**	**Year**	**Malignant (mm)**	**Benign (mm)**
Kanno et al. [10]	2010	25.8 ± 4.1^a^	3.9 ± 3.5^a^
Kobayashi et al. [12]	2012	16.4 (10 to 35)^b^	4.3 (0 to 15)^b^
Zhang et al. [13]	2011	13 (3 to 32)^b^	5 (2 to 7)^b^
Kawada et al. [20]	2014	15 ± 8^a^	6 ± 5^a^

^a^Means ± SD. ^b^Median (range).

## Conclusions

Preoperative prediction of malignancy for branch duct IPMNs is still difficult. Therefore, further studies, including advances in high-resolution imaging or improved molecular-biological techniques, would be required for correct and differential diagnosis of IPMA and IPMC.

## Consent

Written informed consent was obtained from the patients for publication of this case report and any accompanying images.
